# Occipitocervical fusion combined with 3-dimensional navigation and 3-dimensional printing technology for the treatment of atlantoaxial dislocation with basilar invagination: A case report

**DOI:** 10.1097/MD.0000000000018983

**Published:** 2020-01-31

**Authors:** Tianyang Yuan, Guoliang Jia, Lili Yang, Derui Xu, Jun Zhang, Qinyi Liu

**Affiliations:** Department of Orthopaedics, The Second Hospital, Jilin University, Changchun, Jilin, China.

**Keywords:** 3D navigation system, 3D print model, basilar invagination, occipitocervical fusion

## Abstract

**Introduction:**

Basilar invagination (BI) is a common deformity in the occipitocervical region. The traditional surgical method of BI is direct transoral decompression followed by posterior decompression and fixation. Posterior-only decompression and fixation have achieved good efficacy in the treatment of BI in recent years, but complications are common due to the operation in the upper cervical vertebra and the medulla oblongata region. Moreover, posterior-only occipitocervical fusion combined with an intraoperative 3-dimensional (3D) navigation system is relatively rare, and reports of this procedure combined with 3D printing technology have not been published. We present a case of BI treated with posterior-only occipitocervical fusion combined with 3D printing technology and 3D navigation system to reduce the risk of surgical complications.

**Patient Concerns:**

A 55-year-old patient with a history of neck pain and numbness of the extremities for 6 years developed a walking disorder for 1 year.

**Diagnoses:**

Atlantoaxial dislocation with BI.

**Interventions:**

The patient underwent posterior-only occipitocervical fusion combined with intraoperative 3D navigation system and 3D printing technology.

**Outcomes:**

The patient's walking disorder was resolved and he was able to walk approximately 100 m by himself when he was allowed to get up and move around with the help of a neck brace. At 6 months postoperatively, the patient reported that the numbness of the limbs was reduced, and he could walk >500 m by himself.

**Conclusion:**

Occipitocervical fusion is one of the established techniques for the treatment of BI. The biggest advantage of the 2 technologies was that it ensured precise implant placement. The advantages of intraoperative 3D navigation systems are as follows: real-time intraoperative monitoring of the angle and depth of implant placement; the best nailing point can be determined at the time of implantation, rather than according to the operator's previous experience; and the extent of screw insertion is visible to the naked eye, rather than being dependent on the “hand feel” of the surgeon. At the same time, the 3D printing technology can be applied to clarify the relationship between blood vessels and bone around the implant to minimize injury to important structures during implantation.

## 1 Introduction

Basilar invagination (BI) is a common deformity in the occipitocervical region. Its pathogenesis is mostly related to the abnormal embryonic development process, and it may also be related to the compensation after atlantoaxial instability. It is mostly manifested by a series of neurological symptoms, including neck pain, limb weakness, and numbness caused by the odontoid process descending backward and upward into the foramen magnum. BI is often associated with atlantoaxial dislocation (AAD), occipitalization of the atlas, atlas dysplasia, odontoid deformity, Chiari malformation, Klippel–Fell syndrome, and other deformities. For the treatment of BI, direct transoral decompression followed by posterior decompression and fixation has been accepted as a standard procedure over the past 3 decades.^[1–3]^

The traditional surgical method can completely relieve spinal cord compression, but combined anterior and posterior surgeries inevitably increase the risk of surgery and cause considerable trauma to patients. In recent years, posterior-only decompression and fixation have achieved good efficacy in the treatment of BI, which can effectively reduce or avoid the occurrence of pulmonary infection and nonunion of soft palate incision through oral surgery.^[4,5]^ Posterior-only occipitocervical fusion combined with an intraoperative 3D navigation system is relatively rare, whereas reports of this procedure combined with 3D printing technology have not been published.

We report herein a case of congenital odontoid disconnection in a patient with ADD who underwent posterior-only occipitocervical fusion combined with 3-dimensional (3D) navigation system and 3D printing technology to ensure precise screw placement of the cervical vertebra, thereby reducing the risk of surgical complications.

## 2 Case report

**Figure 1:**
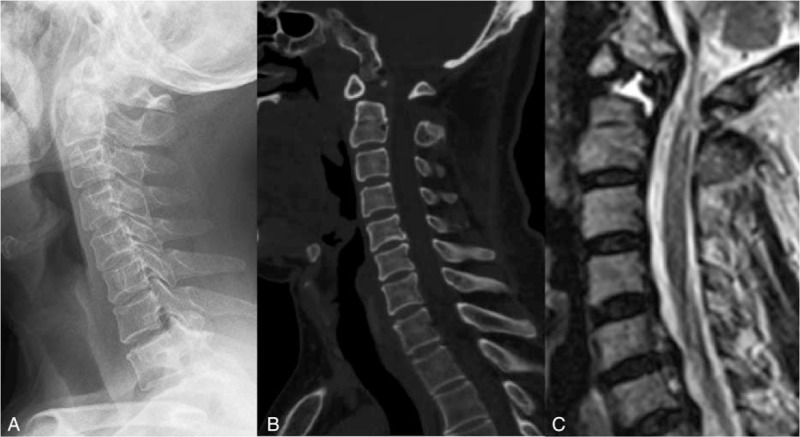
The atlantoaxial instability resulted in mild atlantoaxial dislocation, as observed on the preoperative X-ray (A) and CT scan (B). (C) MRI showed that the spinal cord was compressed by the posterior atlas arch. CT = computed tomography, MRI = magnetic resonance imaging.

A 55-year-old male patient with a history of neck pain and numbness of extremities for 6 years presented with hypoesthesia below the inguinal plane. The right upper extremity numbness was heavier than the left. The abovementioned symptoms were aggravated for 1 year resulting in a walking disorder. The patient required crutches when walking, and the maximum distance that he could walk was 50 m. Physical examination showed grade III strength of each muscle in both upper extremities, grade II grip strength, and grade IV strength of each muscle in both lower extremities. Bilateral Hoffman tests were positive, whereas the tendon reflex of both lower extremities was hyperactive. Magnetic resonance imaging (MRI) of the cervical vertebra showed that abnormal odontoid in the occipitocervical junction area and severe surrounding hydrops. The atlantoaxial instability resulted in mild AAD, as detected via computed tomography (CT), and the spinal cord was compressed by the posterior atlas arch (Fig. 1).

**Figure 2:**
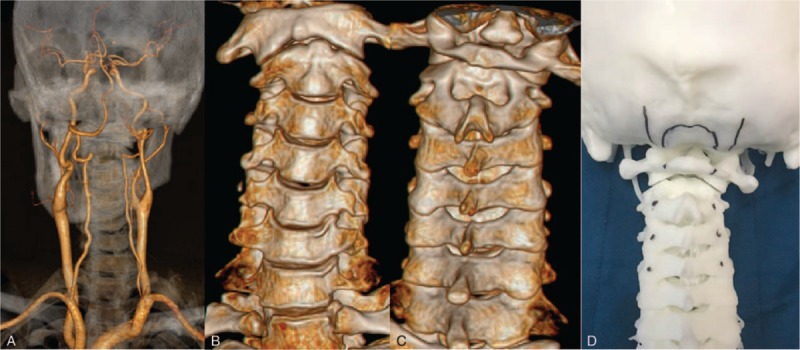
(A) Preoperative cervical CTA to evaluate the relationship between the important blood vessels and bone in the cervical area. (B and C) 3D CT of the cervical was performed to evaluate the bony structure. (D) A 3D printing model of the patient's occipitocervical area was constructed using CTA and 3D-CT. 3D = 3-dimensional, CTA = computed tomography angiography.

After failure of conservative treatment, we decided to perform surgical treatment on the patient to address the walking disorder. Preoperatively, we performed cervical CT angiography (CTA) to evaluate the relationship between the important blood vessels and bone in the cervical area. At the same time, 3D CT of the cervical region was performed to make a 3D print model of the patient's occipitocervical area (Fig. 2). We simulated the depth and angle of cervical screw insertion according to the 3D print model, and finally determined the most suitable cervical screw for the patient. After adequate preoperative preparation, we performed posterior-only occipitocervical fusion combined with intraoperative 3D navigation system.

### 2.1 Operative technique

**Figure 3:**
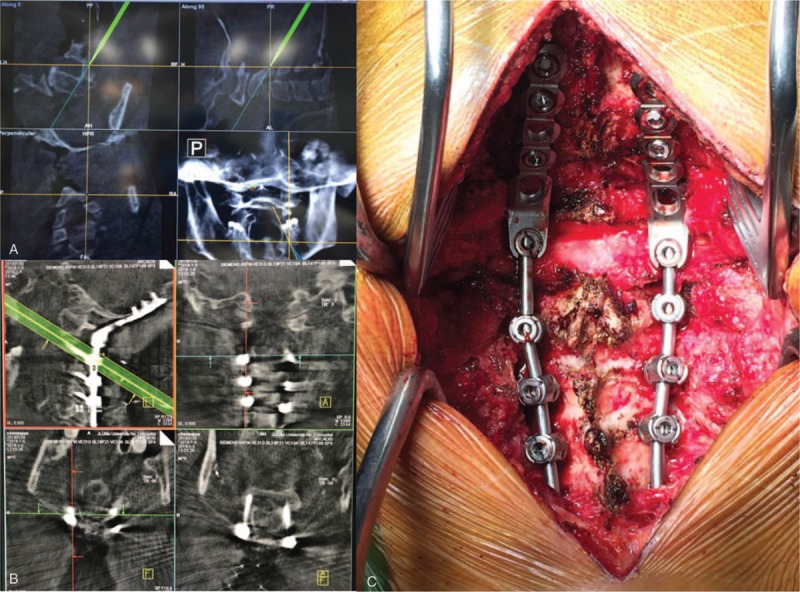
(A) Intraoperative real-time monitoring the angle and depth of implant placement using a 3D-navigation system. (B) The pedicle screws (C2) and mass screws (C3-C4) were in the correct position with the guidance of the 3D-navigation system. (C) The blood supply of dural sac at C1 was well, indicating good decompression. 3D = 3-dimensional.

**Figure 4:**
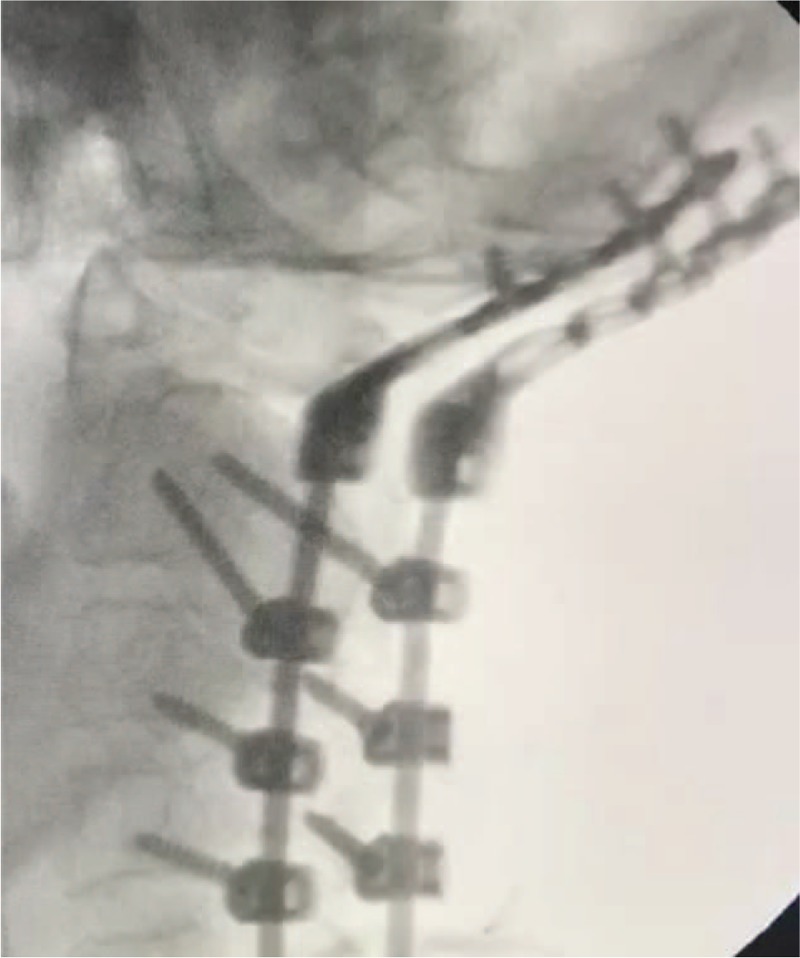
Intraoperative X-ray showing that the screws are in the correct position without displacement.

After induction of general anesthesia, the patient was placed in a prone position. The vertebrae were exposed through an approximately 25-cm longitudinal midline skin incision from the occipital tuberosity to the spinous process of C6. The skin, subcutaneous tissue, ligaments, and deep fascia were exposed layer by layer. The occipital to the C5 spinous processes were fully exposed. The 3D navigation patient tracker was placed at the C5 level, and C-arm scan of the cervical vertebra with preoperative CT imaging after matching was performed. After matching to obtain high quality images, the navigation device was along the left side of the C2 articular process to determine the nail's insertion point, angle, and depth. Pedicle screws were placed at the C2 level on the left, and pedicle screws were placed at the right side of C2 vertebra in the same manner; then, the lateral mass screws were placed at the sides of C3 and C4. The pre-bent connecting rod was inserted into the screw tail groove and the top wire was locked. Three cranial screw casings were inserted into the left connecting rod. The position and angle of the casings were adjusted. Three cranial screws were inserted into the screw holes, and the right cranial base was manipulated in the same manner. The 3 cranial screw casings and screws were then inserted. The positional depth of the pedicle screw was determined to good by c-arm fluoroscopy. The posterior arch edge was removed by grinding and drilling on the right side of the posterior arch of the atlas, and part of the bone was removed by laminar forceps. With the hook nerve dissection, the dural sac was separated from the posterior arch of the atlas, and the posterior arch of the atlas was removed, thereby fully exposing the spinal cord pulses. The spinal cord surface is covered with thickened fibrous scar bands, which are carefully removed (Fig. 3). The spinal cord was covered with a gelatin sponge. The trimmed autogenous cancellous bone and allogeneic artificial bone were implanted on the polished bone surface. After the implantation was confirmed, the neck muscles were restored, and a negative pressure drainage box was retained and fixed. The pedicle screw system was in the right position without displacement, which was confirmed on the intraoperative and postoperative X-ray films (Fig. 4).

**Figure 5:**
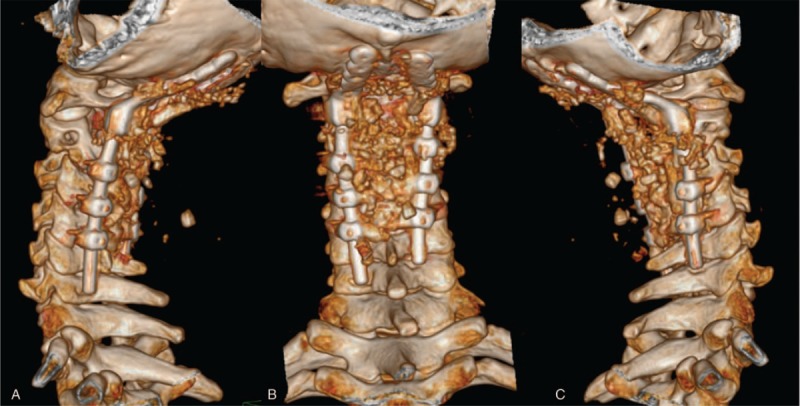
(A and C) After removal of the stitches, 3D-CT was performed and showed that the position of the pedicle screw was not significantly changed from that of the X-ray obtained 2 wk prior. 3D = 3-dimensional, CT = computed tomography.

Owing to the operation in the upper cervical vertebra and the medulla oblongata region, the patient's respiratory and circulatory systems were very likely to be damaged. After the operation we managed to keep the patient in the state of endotracheal intubation for 24 hours and then slowly come out of the anesthesia, and the patient's vital signs were closely monitored. The patient's vital signs were stable when he woke up, and he was conscious and had good limb movements. His grip strength increased from grade II before surgery to grade IV after surgery. Dexamethasone 5 mg/bid was administered twice a day for 5 days. Neurotrophic and anti-infective therapies were given at the same time. The wound healed at the first stage, and the stitches were removed at 14 days postoperatively. After removal of the stitches, 3D-CT was performed, which showed that the position of the pedicle screw was not significantly changed from that of the X-ray obtained 2 weeks prior (Fig. 5). Postoperative MRI was not included in this paper due to the severity of the artifacts. At 3 weeks postoperatively, the patient's walking was ability improved and he was able to walk approximately 100 m by himself when he was allowed to get up and move around with the use of a neck brace. At 6 months postoperatively, the patient reported that the numbness of the limbs was reduced, and he was able to walk >500 m by himself. Physical examination showed grip strength of grade IV, and the rest of the limb muscle strength was grade IV. The tendon reflexes of both lower limbs were normally elicited without hyperactivity. In the past 6 months, there were no other discomforts and related complications, indicating that the operation was successful.

## 3 Discussion

In the past few decades, anterior transoral release or odontoidectomy combined with posterior decompression and fixation has been recognized as the gold standard for the treatment of BI.^[6]^ Previous literature has suggested that, in patients with spinal cord compression, only decompression can effectively solve the patient's neurologic symptoms,^[7]^ but for the treatment of BI, it not only should ease or even alleviate the patient's neurologic symptoms, but also should restore the stability of the occipitocervical area. Thus, for BI patients with nerve compression, posterior fixation and fusion should be performed.^[7–13]^ Traditional combined anterior and posterior surgery can completely relieve spinal cord compression, but it also inevitably increases the risk of surgery and causes considerable trauma to patients. Anterior transoral surgery can cause nonunion of the soft palate incision and wound infection,^[14,15]^ and if combined with posterior fixation surgery, it may cause severe complications such as vertebral artery injury and cerebrospinal fluid leakage. For the patient in this report, the abnormal development of the odontoid led to AAD, and the posterior arch of the atlas compressed the spinal cord. According to the preoperative imaging examination, there is no obvious compression factor on the ventral side of the spinal cord. Combined with this factor, we decided to perform occipital and cervical fusion decompression with the posterior approach only.

In recent years, the posterior-only occipitocervical fusion and decompression have achieved good results in the treatment of BI, which can effectively reduce or avoid the occurrence of nonunion of soft palate incision and pulmonary infection through oral surgery. Yang et al^[5]^ investigated the application of posterior traction combined with occipitocervical fusion in the treatment of BI caused by ADD. They reported the decompression of posterior arch of the atlas and partially removed the margin of the foramen magnum, and the C2 and C3 bilateral lateral mass screws were inserted. The author applied the traction reduction technology and avoid the anterior transoral odontoidectomy and this method achieved good results. In our case, we also removed the posterior atlas arch of the patient and partially removed the margin of the foramen magnum. However, we inserted the pedicle screws into the C2 vertebra and lateral mass screws into the C3 and C4 vertebrae, which would increase the stability, but it was more risky. Since there was no obvious ventral compression in our patient, and the nerve compression was mainly in the posterior atlas arch, the nerve compression was relieved after the removal of the posterior atlas arch and partial removal of the margin of the foramen magnum. In our case, emphasis was placed on the placement of pedicle screws at the C2 vertebral body and the C3 and C4 lateral mass screws. Occipitocervical fusion is associated with a variety of complications, including spinal cord and nerve root damage, vertebral artery injury, dural tears, cerebrospinal fluid leakage, instrumentation failure, inability to achieve bony fusion, and surgical infection.^[16]^ If these complications occur during the screw placement process, the consequences are unimaginable.

Zhu et al^[17]^ reported a case of posterior-only occipital neck fusion in 2018, in which a patient developed a posterior fossa epidural hematoma and died. This patient underwent the procedure without the use of any intraoperative navigation technology, instead the patient was operated solely on the basis of preoperative imaging evaluation combined with the experience of the operator. This case report illustrates the importance of precise screw placement, and even a slight mistake will bring very serious consequences.

In 2018, Linkai et al^[16]^ reported a retrospective analysis of 30 cases treated with occipitocervical fusion combined with o-arm and intraoperative 3D navigation technology. One of the 30 patients had nondominant vertebral artery injury and another patient had implant loosening. Intraoperative navigation was useful in delineating the bony anatomy, but it was not useful in understanding the adjacent vascular or spinal cord anatomy. This disadvantage may be the main factor of vertebral artery injury in these previous reports. The reason for loosening of the implant is that the surgeon in these reports only judged the implant size of the patient through preoperative imaging examination and experience. For our patient, we applied the intraoperative 3D navigation technology to ensure precise implant placement to avoid complications. In our case, we performed detailed preoperative imaging examinations using cervical CTA, cervical 3D-CT, and so on. Then, we constructed a 3D print model of the patient's occipitocervical area at the same time to determine the implant insertion site. This technique effectively evaluated the relationship between blood vessels and bone in patients and minimized the incidence of related complications.

For the treatment of BI, if there are compression factors on the ventral side of the spinal cord, anterior transoral decompression is the preferred treatment. Moreover, posterior fusion fixation is also needed to maintain the stability of the occipital neck. For patients without ventral spinal cord compression, most of the symptoms are related to the instability of the occipital neck; thus, posterior-only decompression and fixation is the primary treatment. Precise screw placement is very important in posterior decompression fixation for occipital neck fusion. Spinal 3D navigation is the best way to ensure greater accuracy of transpedicular screw insertion.^[18]^The advantages of intraoperative 3D navigation technology are as follows: real-time intraoperative monitoring of the angle and depth of implant placement; the best nailing point can be determined at the time of implantation, rather than according to the operator's previous experience; and the extent of the screw insertion is visible to the naked eye, rather than being dependent on the “hand feel” of the surgeon. The abovementioned advantages can ensure optimal accuracy and safety of surgery. However, the 3D navigation technology is not perfect, and it does not provide surgeons with a good observation of the surrounding vascular structure. Thus, in this report, we used 3D printing technology to evaluate the relative relationship between the patient's bone and blood vessels preoperatively, thereby allowing us to determine the size of the implant. The combination of these 2 techniques can maximize the accuracy of occipitocervical fusion.

In conclusion, the postoperative neurological function of our patient recovered significantly, indicating that the combination of 3D printing technology and 3D navigation system for the treatment of BI is effective, is accurate, and has a very high success rate.

## Acknowledgment

The authors would like to thank Editage [www.editage.cn] for English language editing.

## Author contributions

**Conceptualization:** Tianyang Yuan, Derui Xu.

**Data curation:** Tianyang Yuan, Guoliang Jia, Lili Yang, Jun Zhang, Qinyi Liu.

**Investigation:** Guoliang Jia, Lili Yang, Jun Zhang.

**Resources:** Qinyi Liu.

**Visualization:** Guoliang Jia, Jun Zhang.

**Writing – original draft:** Tianyang Yuan.

**Writing – review and editing:** Tianyang Yuan, Jun Zhang, Qinyi Liu.
